# Cancer of Unknown Primary With Squamous Cell Carcinoma Phenotype Presenting as Isolated Axillary Mass

**DOI:** 10.7759/cureus.80094

**Published:** 2025-03-05

**Authors:** Nicole Liang, Mohamed Alshal, Lynne J Goebel

**Affiliations:** 1 Internal Medicine and Geriatrics, Joan C. Edwards School of Medicine at Marshall University, Huntington, USA; 2 Pathology, Joan C. Edwards School of Medicine at Marshall University, Huntington, USA

**Keywords:** axillary lymphadenopathy, cancer of unknown primary, confounding diagnosis, cup, metastatic squamous cell carcinoma axilla

## Abstract

Cancer of unknown primary (CUP) is a rare metastatic malignancy where no primary tumor can be found. We report the case of a 71-year-old female with a strong family history of breast cancer presenting with isolated axillary lymphadenopathy after a tick bite. A biopsy of the axillary mass revealed poorly differentiated squamous cell carcinoma and after extensive evaluation, she was diagnosed with metastatic squamous cell carcinoma with unknown primary tumor. We present this case because of the role of confounding history complicating her initial diagnosis and bringing awareness to a rare disease.

## Introduction

Cancer of unknown primary (CUP) is a rare malignant neoplasm histologically defined as metastatic, yet no primary tumor is found despite clinical examination and imaging studies [1]. The illness more often occurs in older adults with a median age of 65 years. CUP makes up 2-5% of all cancer diagnoses and has a poor median survival of 6-10 months [2]. Over 60% of cases present as adenocarcinomas, while only 5% present as squamous cell carcinomas [3].

CUP progression is difficult to predict due to the heterogeneous nature of the disease. Most cases of CUP exhibit the following common behavior, including an aggressive nature, early metastasis with atypical course, and poor chemotherapy response [2]. The primary lesion is not identified in 20%-50% of cases, even on autopsy [4]. We present a case of metastatic squamous cell carcinoma in the axillary lymph nodes with no apparent primary to bring awareness to a rare disease and to note how confounding information in her history complicated the clinical reasoning to arrive at the true diagnosis.

## Case presentation

A 71-year-old female never-smoker with a strong family history of breast cancer (mother and sister in their early 60s) presented to her primary care physician with pain in her left axilla and breast. She suffered a tick bite on her left arm two weeks prior and received prophylactic doxycycline. Physical examination revealed a 2 cm mildly tender, firm mass in the left axilla with no skin lesions, rashes, or breast masses. There were no other swollen glands, and the rest of her physical exam was normal. Her primary care physician considered a diagnosis of Lyme Disease and ordered a chest X-ray, complete blood count, and Lyme antibody titers to further evaluate other causes of lymphadenopathy. The patient’s history is significant for a 0.4 cm x 0.7 cm primary basal cell carcinoma on the right upper arm approximately one year previous for which she had Mohs surgery with negative superficial and deep surgical margins and no evidence of recurrence. She did not have any immunosuppression, a history of exposure to excessive ultraviolet radiation, or occupational exposures that would increase her risk of cancer. She did not smoke or drink alcohol. Her family history was negative for other cancers including ovarian and colon cancer and the results of BRCA (Breast Cancer Gene) testing on her mother or sister were not known. 

The patient told the oncology consultant that she first noticed the axillary mass eight months earlier but this information was not obtained by the primary care physician. The mass worsened after the tick bite prompting her to seek medical attention. The patient felt well during the visit, with no reported chest pain, dyspnea, headache, bone pain, or unintentional weight loss. She denied seeing any new skin lesions since the basal cell carcinoma on the opposite arm from the previous year. 

**Figure 1:**
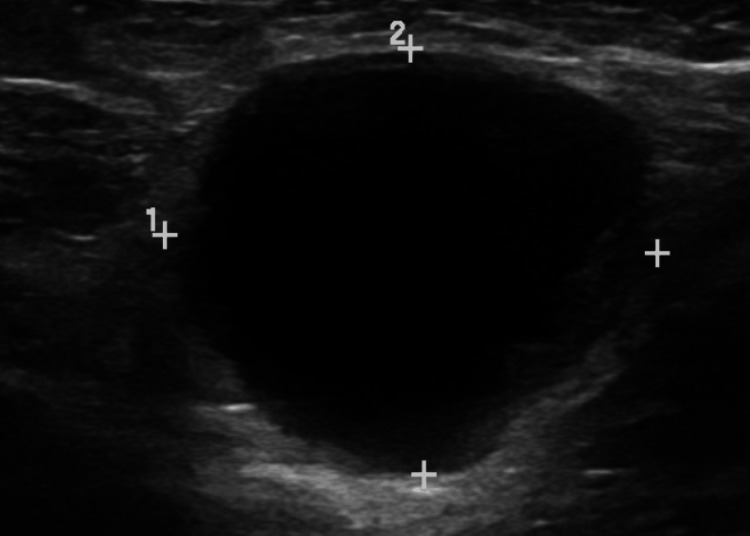
Ultrasound of left axillary mass performed on LOGIQ S8. Measurement 1=2.62 cm, measurement 2=2.27 cm.

The chest X-ray and complete blood count showed no sign of infection or tumor, and antibodies against Lyme disease were negative. The patient underwent digital mammography with a reading of breast imaging-reporting and data system (BI-RADs) 4 indicating a suspicious finding but not definitively malignant. The breasts had scattered areas of fibroglandular density, but the left axilla contained a hyperdense mass. Ultrasound revealed a suspicious 2.62 cm x 2.27 cm hypo-echoic lesion containing peripheral nodules and internal septal lines (Figure 1).

The breast surgeon ordered magnetic resonance imaging (MRI) of both breasts to look for possible occult primary breast cancer since her mother and sister had breast cancer in their early sixties. The breast MRI revealed no masses or suspicious areas of enhancement. There were no additional abnormal lymph nodes. 

**Figure 2:**
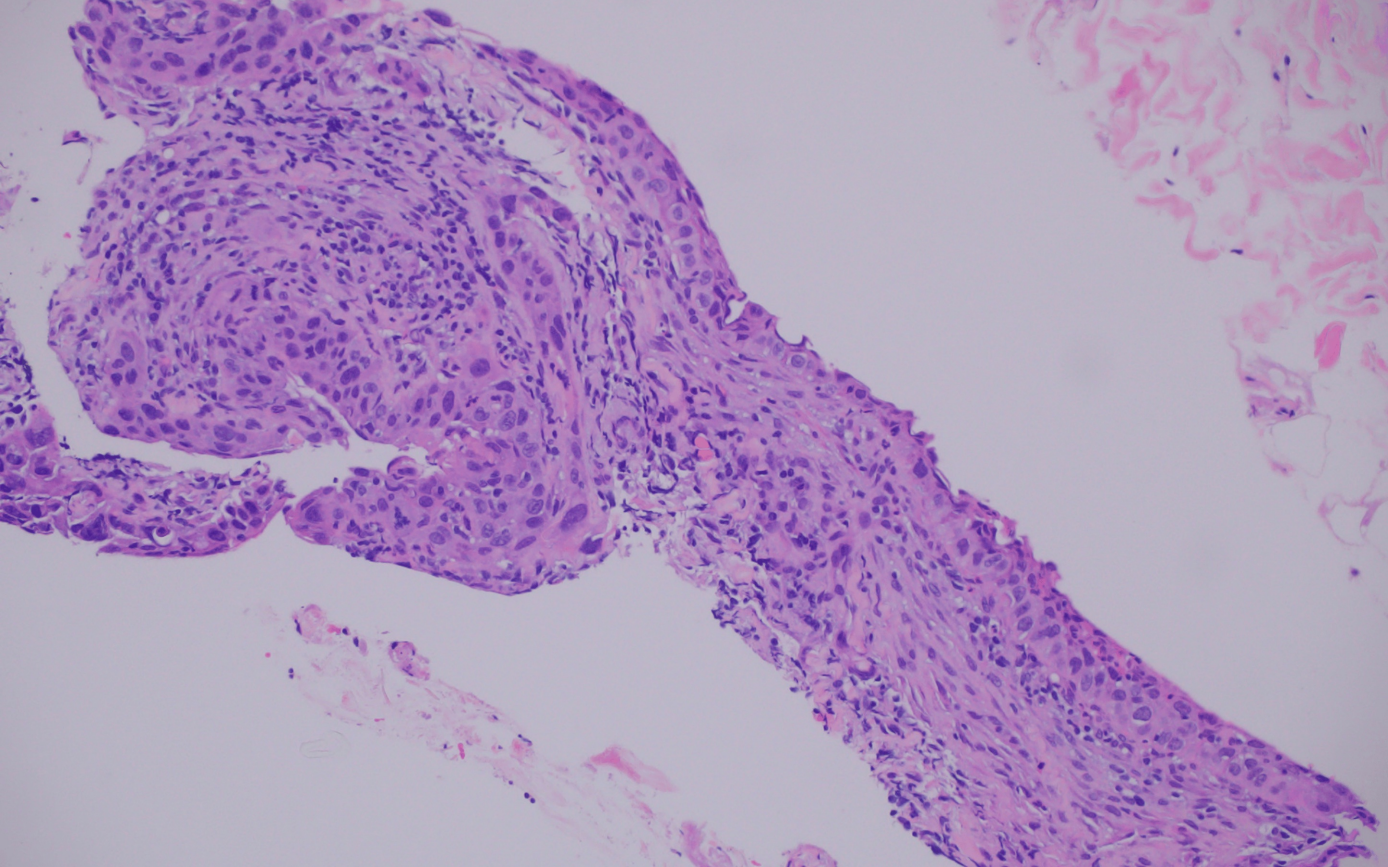
Histologic examination demonstrates poorly differentiated squamous cell carcinoma (Hematoxylin and Eosin stain, original magnification X200)

Histologic examination of a core needle biopsy of the axillary mass revealed poorly differentiated squamous cell carcinoma involving lymphoid and fibro adipose tissue composed of large epithelial cells with nuclear pleomorphism, eosinophilic cytoplasm, and prominent nucleoli (Figure 2).

**Figure 3:**
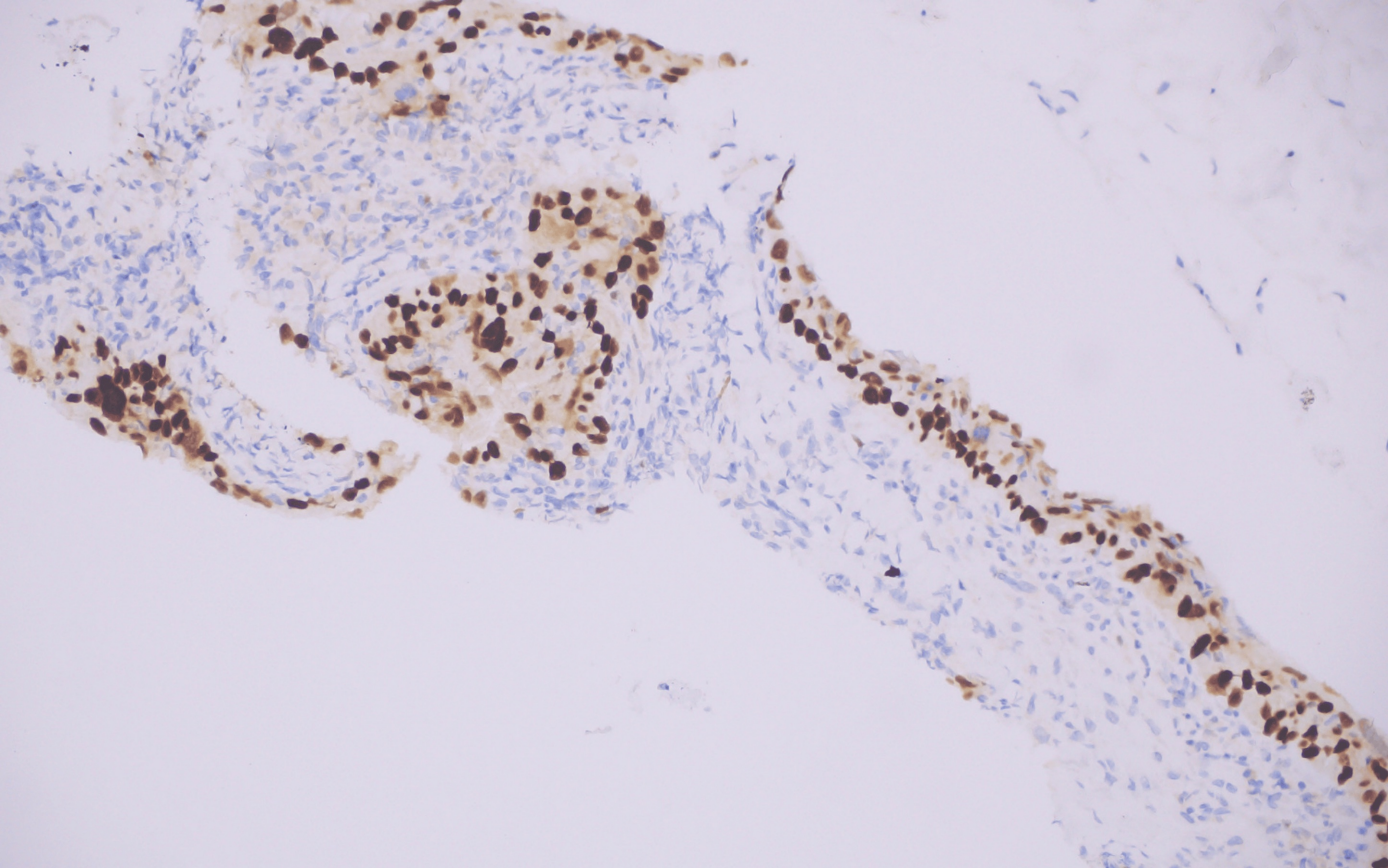
The tumor cells are positive for p63 stain, confirming squamous differentiation

Immunohistochemistry staining was positive for cytokeratin AE1/AE3, tumor protein 40 (p40), and tumor protein 63 (p63) (Figure 3), while negative for S100 (protein soluble in 100% saturated ammonium sulfate), mammaglobin, GATA binding protein 3 (GATA3), sex-determining region y related high mobility group box containing proteins 10 (SOX10), tumor protein 16 (p16), cytokeratin 7 (CK7), and cytokeratin 20 (CK20).

**Figure 4:**
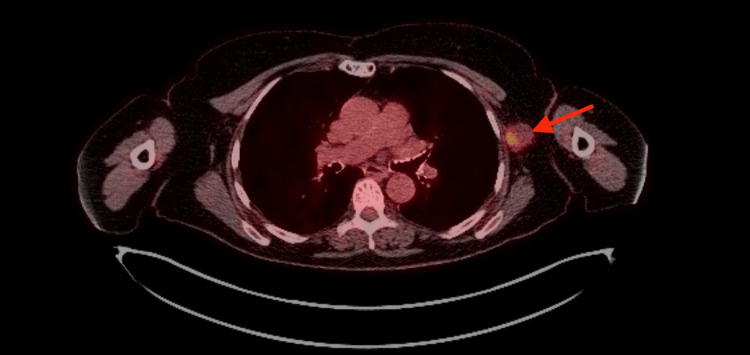
PET scan reveals axillary uptake (arrow) with maximum SUV (standardized uptake value) of 6.3.

Overall, these findings are consistent with poorly differentiated squamous cell carcinoma. Physicians searched for the site of the primary tumor by obtaining a full-body positron emission tomography (PET) scan. However, this only showed the hypermetabolic left axillary lymph node concerning for malignancy (Figure 4).

There were no other hypermetabolic lesions or evidence of distant metastasis. The patient underwent treatment for stage 3 metastatic squamous cell carcinoma. She underwent two rounds of cemiplimab infusions followed by lymphadenectomy. Pathologic evaluation of the lymph node found no evidence of malignancy following immunotherapy, and no PET avid lesions were detected on follow-up. The patient is cancer-free at six-month follow-up. She is being followed at a tertiary care center every three months.

## Discussion

Our patient presented with an isolated enlarged lymph node in the axilla and was eventually diagnosed with cancer of unknown primary (CUP) squamous cell carcinoma phenotype. The differential diagnosis of isolated axillary adenopathy is broad, encompassing both benign and malignant etiologies. Benign causes commonly include non-specific reactive follicular hyperplasia secondary to local infections, insect bites, or trauma, cat-scratch lymphadenitis, dermatopathic lymphadenopathy, sinus histiocytosis, granulomatous disease (such as sarcoidosis), autoimmune causes such as systemic lupus erythematosus and rheumatoid arthritis, and rare benign heterotopic epithelial inclusions such as intranodal cystic epithelial inclusions or intranodal heterotypic mammary ducts [5,6]. Malignant etiologies may include primary lymphoma and metastatic carcinoma, most commonly originating from the breast, lung or skin [6].

Lymphadenopathy after a tick bite initially led to an incorrect diagnosis. The doctor heard “tick bite and axillary lymphadenopathy” and having seen this previously, thought Lyme disease was the most likely diagnosis. In the face of a negative test for an infectious cause, physicians should have a high index of suspicion for occult malignancies, especially in elderly patients. In this patient, the diagnosis was further complicated due to her strong family history of breast cancer. As a result, she was referred to a breast surgeon and had breast imaging. This case highlights how the use of heuristics in clinical decision-making can sometimes be misleading. Heuristics allow physicians to look at data and quickly make connections to arrive at the most likely diagnosis. While these shortcuts can help make rapid diagnoses in an emergency setting, they may also cause physicians to overlook contrary evidence and forgo in-depth testing to reach the correct diagnosis. It is crucial to maintain diagnostic flexibility and explore all potential differentials until the correct diagnosis is made to ensure optimal patient care [7].

Diagnosing CUP requires extensive clinical workup to determine the location of the primary tumor. Sometimes, lesions are discovered on routine testing such as mammograms or colonoscopies [3]. Physical examination, blood tests, and radiological studies such as chest X-rays are common first steps. Further testing to determine the site of origin can include magnetic resonance imaging or positron emission tomography scans, as with our patient [2].

The etiology of CUP is currently unestablished. CUP could be the metastasis from a small, regressing, or otherwise undetectable primary tumor. This may be why most primary tumors are not identified despite comprehensive imaging. CUP may also be its own early-disseminating metastatic entity with no primary tumor development. Treatment of metastatic tumors relies heavily on primary tumor characteristics. Without this information, CUP may require a different approach than typical metastatic tumors [8]. Epigenetic alterations, chromosomal rearrangements, and greater tumor mutation are associated with a worse prognosis [9]. Smoking increases the risk of CUP, and suggestive evidence links alcohol and diabetes mellitus [10]. There may be a familial component, as familial clustering studies found an increased risk of CUP among close relatives [2]. Our patient did not have any of these risk factors.

Overall, the CUP prognosis is poor, with 1-year survival hovering around 20% [2]. Metastatic squamous cell carcinoma (SCC) with unknown primary, our patient’s diagnosis, is associated with a higher 1-year survival probability of approximately 36% [1]. Many cases of metastatic SCC of unknown origin metastasize to cervical lymph nodes, often from a primary head and neck cancer associated with Human Papilloma Virus (HPV) infection [3]. Metastasis to the axillary lymph node is a rare and poorly characterized presentation, potentially associated with a primary lung or skin neoplasm. A retrospective review of all patients seen over a 25-year period at Memorial Sloan Kettering Cancer Center (MSKCC) revealed only 12 cases of metastatic SCC of unknown primary presenting as axillary lymphadenopathy [11]. Based on guidelines from the Spanish Society of Medical Oncology, metastatic SCC is divided into favorable and unfavorable groups, with the unfavorable group (80% of cases) showing high tumor burden and poor prognosis. Our patient falls into the favorable group as she only had a single, resectable metastasis which is associated with a better outcome [12]. 

Immunohistochemistry (IHC) on biopsy specimens is crucial for determining the tissue of origin. Broad-spectrum cytokeratins, such as AE1/AE3, confirm epithelial lineage. S100 and SOX10 negativity excludes melanoma [13]. In our patient, tumor cells demonstrated positive expression for tumor p40 and p63 (p40 being more specific than p63), which is highly specific for squamous differentiation [14]. P16 serves as a surrogate marker for HPV infection, aiding in the sub-classification of SCC, particularly from oropharyngeal origin [2].

The expression patterns of CK7 and CK20, when used in conjunction, can provide further diagnostic clues, aiding in the identification of primary tumor sites. Peridiaphragmatic gastrointestinal organs such as the pancreas, biliary tree, stomach, and urinary bladder are typically CK7+/CK20+. Merkel cell or colorectal carcinomas are usually CK7-/CK20+. In contrast, CK7+/CK20- carcinomas typically originate from above the diaphragm (lung, breast, thyroid, salivary gland) or in the female genital tract. Double negative (CK7-/CK20-) tumors, as in our patient, can arise from the liver, kidney, prostate, germ cell tumors, adrenal gland, squamous cell carcinomas, and most neuroendocrine carcinomas. Common site-specific markers are essential in distinguishing tumors of diverse origin, including caudal homeobox 2 (CDX2) for gastrointestinal origin, GATA3 for breast and urothelial origin, paired box 8 (PAX8) for thyroid, kidney, and ovary origin, and thyroid transcription factor 1 (TTF-1) for lung and thyroid origin [2]. Mammaglobin serves as a marker of breast-origin tumors. This panel is particularly useful in cases involving metastatic adenocarcinoma or urothelial carcinoma with extensive squamous differentiation. 

Molecular profiling may be helpful in determining the location of the primary tumor. Molecular cancer classifier assays (MCCAs) are specialized assays performed to determine the tissue of origin. MCCAs detect site-specific gene expression profiles using RT-PCR (Reverse Transcriptase-Polymerase Chain Reaction), testing 92 different genes potentially expressed by the tumor. This assay is distinct from comprehensive molecular profiling done on most cancers to determine mutations. It can predict primary tumor sites correctly in approximately 80% of cases. There are limited reports of this technology being used on SCC, but it may be an effective diagnostic tool in the future [15].

Of particular interest is the patient’s previous basal cell carcinoma in the opposite arm. This tumor is likely unrelated to the axillary lesion since the conversion of cell type from basal cell to squamous cell is not reported. Previous literature associating CUP cases with a prior cancer diagnosis is sparse. Matylevich et al.. described a case of metastatic retroperitoneal SCC of unknown primary with prior endometrial cancer. The CUP was not attributed to the previous cancer as it was a different cell type, adenocarcinoma, and not a squamous cell [16]. The previously mentioned MSKCC study on metastatic SCC of unknown primary revealed 55% of patients had a prior history of cancer. However, the sample size was small with only twenty in the study group [11]. It remains uncertain if there is merit in investigating a correlation between CUP diagnosis and previous cancer history.

Treatment of CUP varies depending on tissue characteristics as determined by imaging and IHC. If local lymph nodes are involved in a site-specific SCC, lymphadenectomy is recommended. Radiotherapy and platinum-based chemotherapies can be concurrently used [17]. The MSKCC study determined that axillary or inguinal lymphadenectomy greatly improved outcomes, with 65% of patients surviving to 5 years after the procedure [11]. Molecular and genomic profiling can guide the administration of targeted therapies, effectively managing disease. Immunotherapy use is promising, especially in CUP tumors with programmed cell death ligand 1 (PD-L1) overexpression [2,18]. A global CUP clinical trial analyzed the benefit of using targeted therapy with immunotherapy vs. conventional chemotherapy. Preliminary results showed significantly increased progression-free survival when molecularly guided therapy was used compared to traditional platinum-based chemotherapy (6.05 months versus 4.40 months, p=0.0079) [19]. In our case, the patient received cemiplimab, a monoclonal antibody against PD-1 on T-cells, prior to lymphadenectomy. It is indicated for metastatic cutaneous SCC uncurable by surgery or radiation [20]. The treatment was effective in our patient, who currently has no PET avid disease at six-month follow-up. She will continue to be followed at three-month intervals for the first two years and then every six months after that. 

## Conclusions

The present case of metastatic squamous cell carcinoma in the axillary lymph nodes with unknown primary origin is a rare disease. The patient’s presentation was confounded by a misleading tick bite and a strong family history of breast cancer, and despite extensive diagnostic workup, a primary site could not be found. Treatment for this type of illness is based on clinical presentation and tumor markers rather than a standardized formula. Further research is required on how this type of cancer develops, the relationship to a personal history of other cancers, better methods of detection of a primary tumor, and evolving treatments to improve patient outcomes.

## References

[1] Krämer A, Bochtler T, Pauli C (2023). Cancer of unknown primary: ESMO clinical practice guideline for diagnosis, treatment and follow-up. Ann Oncol.

[2] Laprovitera N, Riefolo M, Ambrosini E, Klec C, Pichler M, Ferracin M (2021). Cancer of unknown primary: challenges and progress in clinical management. Cancers (Basel).

[3] (2025). UpToDate: squamous cell carcinoma of unknown primary. https://www.uptodate.com/contents/squamous-cell-carcinoma-of-unknown-primary-site.

[4] Pentheroudakis G, Greco FA, Pavlidis N (2009). Molecular assignment of tissue of origin in cancer of unknown primary may not predict response to therapy or outcome: a systematic literature review. Cancer Treat Rev.

[5] Resetkova E, Hoda SA, Clarke JL, Rosen PP (2003). Benign heterotopic epithelial inclusions in axillary lymph nodes. Histological and immunohistochemical patterns. Arch Pathol Lab Med.

[6] Gaddey HL, Riegel AM (2016). Unexplained lymphadenopathy: evaluation and differential diagnosis. Am Fam Physician.

[7] Whelehan DF, Conlon KC, Ridgway PF (2020). Medicine and heuristics: cognitive biases and medical decision-making. Ir J Med Sci.

[8] Conway AM, Mitchell C, Kilgour E, Brady G, Dive C, Cook N (2019). Molecular characterisation and liquid biomarkers in carcinoma of unknown primary (CUP): taking the 'U' out of 'CUP'. Br J Cancer.

[9] Bochtler T, Wohlfromm T, Hielscher T (2022). Prognostic impact of copy number alterations and tumor mutational burden in carcinoma of unknown primary. Genes Chromosomes Cancer.

[10] Hermans KE, Kazemzadeh F, Loef C, Jansen RL, Nagtegaal ID, van den Brandt PA, Schouten LJ (2023). Risk factors for cancer of unknown primary: a literature review. BMC Cancer.

[11] Wach MM, van Beek E, Ayabe R (2018). Metastatic squamous cell carcinoma of known and unknown primary origin treated with axillary or inguinal lymphadenectomy. Am J Surg.

[12] Losa F, Fernández I, Etxaniz O (2022). SEOM-GECOD clinical guideline for unknown primary cancer (2021). Clin Transl Oncol.

[13] Beauchamp K, Moran B, O'Brien T, Brennan D, Crown J, Sheahan K, Cotter MB (2023). Carcinoma of unknown primary (CUP): an update for histopathologists. Cancer Metastasis Rev.

[14] Affandi KA, Tizen NM, Mustangin M, Zin RR (2018). p40 Immunohistochemistry is an excellent marker in primary lung squamous cell carcinoma. J Pathol Transl Med.

[15] Greco FA, Lennington WJ, Spigel DR, Hainsworth JD (2015). Poorly differentiated neoplasms of unknown primary site: diagnostic usefulness of a molecular cancer classifier assay. Mol Diagn Ther.

[16] Matylevich OP, Kurchankou MA, Kopsсhaj PA, Schmeler KM (2024). HPV-related metastatic retroperitoneal pelvic squamous cell carcinoma of unknown primary origin in a patient previously treated for endometrial cancer. Int J Surg Case Rep.

[17] Zhu QZ, Li HJ, Li YQ, Yu XH, Shu KY (2023). Pelvic metastatic squamous cell carcinoma of unknown primary site: A case report and brief literature review. Medicine (Baltimore).

[18] Zhang M, Zhao M, Jin LF, Shen WZ (2021). Successful treatment using immunotherapy in combination with chemotherapy for metastatic squamous cell carcinoma of unknown primary origin with bulky abdominal mass: a case report. Medicine (Baltimore).

[19] (2024). NIH: A phase II randomized study comparing the efficacy and safety of targeted therapy or cancer immunotherapy versus platinum-based chemotherapy in patients with cancer of unknown primary site (CUPISCO). https://clinicaltrials.gov/study/NCT03498521.

[20] Lee A, Duggan S, Deeks ED (2020). Cemiplimab: a review in advanced cutaneous squamous cell carcinoma. Drugs.

